# The Impact of Government-Led Farmland Construction on Market-Oriented Farmland Transfer—Evidence from Shandong, China

**DOI:** 10.3390/ijerph20043701

**Published:** 2023-02-19

**Authors:** Hongkun Ma, Hao Zhu, Shuhan Ren, Rudi Liu, Cuixia Qiao

**Affiliations:** 1 School of Economics, Shandong Normal University, Jinan 250358, China; 2 Academy for County Economy Research, Shandong Normal University, Jinan 250358, China

**Keywords:** high-standard farmland construction, farmland transfer, fragmentation, labor transfer, binary probit model

## Abstract

This study explored the impact of government-led high-standard farmland construction (HSFC) on market-oriented farmland transfer using a unified analysis framework of HSFC and farmland transfers. We used a binary probit model based on 660 questionnaires from five counties in Shandong Province, China to empirically analyze this impact. The results show that HSFC can significantly promote farmland lease-in while inhibiting lease-out. We found that farmland fragmentation plays a significant role in moderating this impact, which is illustrated by the fact that improved farmland fragmentation does not promote HSFC in the context of farmland lease-in. Furthermore, it can effectively alleviate the inhibitory effect of HSFC on farmland lease-out. The impact of HSFC on farmland transfer has significant labor transfer heterogeneity. For households with a low degree of labor transfer, HSFC can significantly promote farmland lease-in and inhibit lease-out, while for households with a high degree of labor transfer, the above effect is not significant.

## 1. Introduction

Promoting high-standard farmland construction (HSFC) and farmland transfers are the two key farmland policies of the Chinese government. The HSFC is led by governments at all levels, including planning, funding, and implementation, with the core goal of improving farmland productivity by strengthening infrastructure construction. By 2022, China had completed 1 billion mu (a unit of area in China, 1 mu = 0.0667 hectares) of high-standard farmland, accounting for approximately 50% of the country’s total farmland. Farmland transfers are highly market-oriented transactions that require managerial authority. They are designed to expand the production area and give full play to economies of scale. In recent years, the Chinese government has introduced several measures to encourage and guide farmers in carrying out farmland transfers to achieve moderate-scale management. As a result, by 2022, China’s farmland transfer rate will exceed 30%.

Government-led HSFC can potentially impact market-oriented farmland transfers. Existing studies show that under the condition of marketization, comparative income can directly and indirectly affect farmers’ willingness to transfer farmland [1,2,3]. Government-led HSFC can improve the quality of farmland [4,5,6], promote agricultural mechanization [7], and enhance productivity [8,9,10]; thus, it can remedy the comparative disadvantage of farming income, strengthening farmers’ willingness to lease in more farmland. However, although the two policies of the HSFC and farmland transfer are crucial to the current development of China’s agriculture, studies have seldom incorporated the two into a unified analytical framework and their potential impact has not been thoroughly studied, let alone confirmed. At present, academic research on the effects of HSFC is mainly interested in the economic effects of promoting grain production [11,12], increasing farmers’ income [13], the social profitability of driving regional development [14], and reducing carbon emissions [15]. As for the factors influencing farmland transfer, existing research has mainly focused on property rights [16,17,18], agricultural socialized services [19], human capital [20], social capital [21], and fragmentation [22]. In 2022, Chen and Hong conducted a study exploring the impact of HSFC on farmland transfer with Jiangxi Province as the sample [23]. Since Jiangxi is a mountainous and hilly province with only 2.1% of the country’s farmland, this necessarily limits the sample size of the study. Meanwhile, Jiangxi’s HSFC completion rate and farmland transfer rate is only 11%, which not only limits the sample size, but also affects the diversity, representativeness, and reference value of samples, and hence weakens the conclusions’ reliability. In short, in order to fully explore the impact of government-led HSFC on market-oriented farmland transfers, it is necessary to select samples in a larger range and unify them in one analytical framework, so as to provide more reliable policy implications for promoting HSFC and farmland transfer.

In light of existing research, this study engaged HSFC and farmland transfer from the perspective of a unified analysis framework. To ensure good sample size and diversity, we selected Shandong Province as our sample province. Shandong Province is a large agricultural province in China with relatively high HSFC completion and farmland transfer rates. To make it convenient to compare with the findings about the influence of HSFC on farmland transfer in Jiangxi Province, the general idea and framework of Chen and Hong [23] were followed. In this study, we first empirically tested the impact of HSFC on farmland transfer using a benchmark regression. Next, we explored the moderating effect of farmland fragmentation on the above impact. Last, we examined the heterogeneity of the above impact under different degrees of labor transfer.

## 2. Hypotheses, Data, and Methods

### 2.1. Research Hypothesis

**Figure 1:**
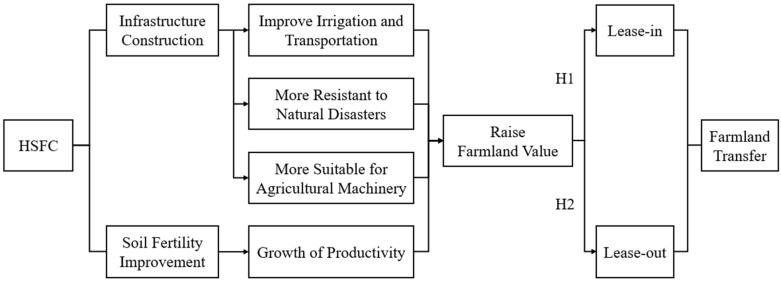
Theoretical Framework of this study.

In 2022, The Ministry of Agriculture and Rural Affairs of China promulgated and implemented the newly revised General Principles for the High Standard Farmland Construction, GB/T30600-2022 (GPHSFC). According to the GPHSFC, the core goal of the HSFC is to significantly improve the productivity of farmland. Specifically, the construction should be carried out in two dimensions: first, the construction of infrastructure in line with the modern agricultural production mode, including field renovation, facilities for water supply and drainage, field roads, and electricity substation; and second, to further improve soil fertility, including soil improvement and the elimination of barrier layers. The construction of farmland that meets the national GB/T30600-2022 standard will effectively improve irrigation and transportation facilities, enhance the ability of farmers to resist natural disasters, reduce losses when natural disasters occur, and ensure the steady growth of production [2,7]. At the same time, constructing a series of infrastructures provides convenience for the application of agricultural machinery and thus alleviates the constraint of a labor shortage in rural labor transfer [8]. The fact that improving irrigation and transportation facilities, enhancing the ability of farmers to resist natural disasters, reducing losses in natural disasters, and ensuring the steady growth of production can raise farmland value has been confirmed by literatures. Zhang and Jiang’s study shows that the lag in the agricultural infrastructure facilities is an important reason for restricting the increase in farmland value [24]; Li and Li took Liangxiang Village of Qingdao as an example and found the farmland value was effectively raised by strengthening the construction of farmland infrastructure [25]; Yan and Cui focused on the value of farmland around the city and confirmed it can be effectively raised by developing the integration of transportation among urban clusters [26]. Using descriptive statistics and the farm value model, Ohajianya, D.O., et al. [27] found that by enhancing the ability of farmers in the Imo State of Nigeria to cope with natural disasters, the production efficiency of local farmland was greatly improved, driving the promotion of local farmland. Obasi’s research in the same region also confirmed that farmland productivity and value can be effectively improved by enhancing the ability of farmers to resist natural disasters [28]. Bhattarai et al. confirmed that the penetration rate of tractors in India has been greatly increased through the continuous efforts of the last several decades, which has enhanced India’s food production capacity and also improved the farmland value [29]. The driving effect of agricultural productivity on the value of farmland has also been fully explored by examples of literature. Its internal mechanism is that when the unit scale of farmland can produce more agricultural products, higher agricultural operating income can be obtained, which will enhance the rarity of farmland [30,31,32,33]. In total, HSFC increases the value of farmland, alleviates the vulnerability of agricultural production, enhances the attraction of farmland to farmers, and strengthens farmers’ willingness to lease in farmland to achieve scale efficiency. Based on this knowledge, we proposed the following research hypothesis on the impact of HSFC on farmland lease-in (Figure 1).

**H1:**  *HSFC can promote farmers to lease in farmland.*

HSFC can effectively improve agricultural productivity, alleviate the impact of farmland on a labor force shortage, and make farmland more attractive to farmers, compared with increasing farmers’ willingness to lease in more farmland. Therefore, HSFC will inhibit farmers’ willingness to lease out. Accordingly, we proposed the following research hypothesis on the impact of HSFC on farmland lease-out (Figure 1).

**H2:**  *HSFC can restrain farmers from leasing out their farmland.*

### 2.2. Data Sources

**Figure 2:**
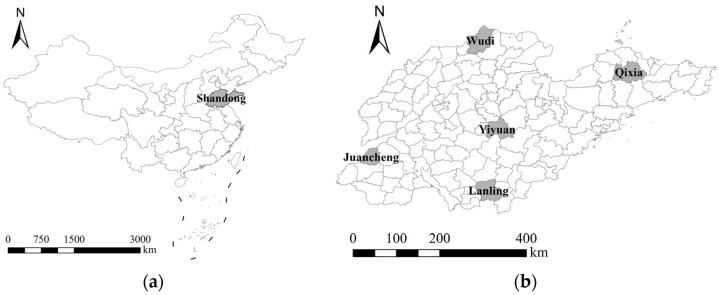
Sample location (**a**) The location of Shandong Province; (**b**) The five randomly selected counties in Shandong Province.

**Figure 3:**
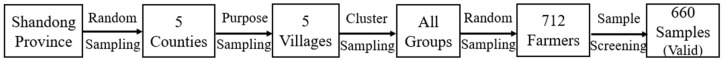
Sampling Processes. Due to the COVID-19 epidemic, some counties and villages in Shandong Province were on lockdown and were automatically excluded from the sampling.

We conducted a household survey in Shandong Province in 2022 to collect data for this study (Figure 2a). Shandong was chosen as a sample province for the following reasons. First, it is a major agricultural province in China with some of the highest rates of high-standard farmland and farmland transfers in the nation (over 69% and 42.6%, respectively). Second, it is also composed of coastal and inland areas distributed in mountains, hills, plains, and other landforms. These factors are conducive to ensuring the diversity and representativeness of the sampled farmers. The survey was divided into four steps (Figure 3). First, to ensure the representativeness of the sample regarding region and topography, Shandong Province was divided into five regions and one county was randomly selected from each region, including Yiyuan (the central), Wudi (northwest), Juancheng (southwest), Lanling (south), and Qixia (northeast), as shown in Figure 2b. These counties are distributed in the plains, mountains, hills, and other landforms. Subsequently, a sample village was selected from each county to further improve representativeness using purposive sampling. These sample villages had their characteristics in scale, industrial structure, style, features, etc. Subsequently, all groups in each sample village were investigated using cluster sampling. Finally, farmers were selected from each village group through random sampling of questionnaires. A total of 712 questionnaires were collected; 660 valid samples were obtained, and 52 invalid ones were eliminated. As a result, the sample efficiency is 92.7%.

### 2.3. Variables

(1) Dependent Variable. The dependent variable was “whether there was farmland transfer.” In this study, farmland transfer comprises farmland lease-in and lease-out. If lease in or lease out occurred, the value was 1; otherwise, it was 0.
(2) Main independent variable. The core independent variable was “whether high-standard farmland has been constructed.” If the village group to which the farmer belonged had carried out HSFC, the value was 1; otherwise, it was 0.
(3) Control Variables. To control the impact of other factors on farmland transfer, we set up a series of control variables from the three dimensions of the village, families, and farmers. For the village, two variables—topography and location—were selected. Regarding families, seven variables were selected: the number of household laborers, the proportion of completely non-agricultural labor, the total scale of farmland, the number of farmland plots, the number of farm machinery, the frequency of utilizing social services, and the expenditure on human relations. Finally, regarding individual farmers, three variables were selected: age, education level, and annual medical expenses.
(4) Fixed effects. By referring to existing research [23], we incorporated the fixed effect of counties into the follow-up model to control the impact of regional heterogeneity on the estimation result.

According to the statistical results, the average value of leasing out farmland for the dependent variable was 0.35. In contrast, leasing in was only 0.09, indicating that farmland transfer in the sample area was not common. In this study, we only count whether there was farmland leasing in or out, without considering the scale and quantity of farmland, which may lead to the inconsistency between the number of farmers leasing out and that of leasing in. For the main independent variable, the average value of whether high-standard farmland has been constructed was 0.69, slightly lower than the average level of 71% in Shandong Province. Overall, no abnormal values were found for any variable, and the degree of dispersion met the need for linear regression (Table 1).

### 2.4. Model Setting

Since the impact of HSFC and other control variables on the dependent variable “whether there was farmland transfer” would be firstly explored and the dependent variable is a Binary discrete 0-1 variable, we constructed a binary probit model. Its basic expression is as follows:

(1) $$LI_{i}=\alpha_{0}+\alpha_{1}HSFC_{i}+\overset{n}{\underset{i=2}{\sum }}\alpha_{i}\varphi_{i}+\delta \;$$

(2) $$LO_{i}=\beta_{0}+\beta_{1}HSFC_{i}+\overset{n}{\underset{i=2}{\sum }}\beta_{i}\varphi_{i}+\varepsilon \;$$

In Equations (1) and (2), $LI_{i}$ and $LO_{i}$ are the farmland lease-in and lease-out of the peasant household, i, respectively; $HSFC_{i}\;$ represents whether the farmland of the peasant household, i, was constructed with high-standard farmland; φ  indicates the control variables; $\alpha_{0}$ and $\beta_{0}$ are constant terms; and δ and ε  are perturbation terms.

## 3. Results

### 3.1. Benchmark Regression

Benchmark regression (Table 2) shows that the HSFC can significantly encourage farmers to lease in farmland and inhibit farmers from leasing out. Regarding farmland leasing in, the impact of HSFC was significantly positive at the 1% level (models 1 and 2), and the coefficients were 0.984 and 0.713, respectively, regardless of whether the control variable was added; these findings further verify H1. On the contrary, HSFC significantly inhibited farmers from leasing out their farmland. Furthermore, the estimated results of models 3 and 4 were significant at the 5% and 10% levels, respectively. Thus, Hypothesis H2 was verified.

Regarding control variables, village location and topography had no significant impact on farmland transfers. Among the seven peasant household variables, while the size of the household labor force has no significant impact, the proportion of entirely non-agricultural labor force significantly inhibited the lease in of farmland at the 10% level. Furthermore, it significantly promoted the lease out of farmland at the 5% level. Comparing the two results above reveals that an increase in the proportion of the non-agricultural labor force decreases the number of labor forces engaged in agricultural production. Therefore, peasant households are reluctant to lease in farmland while leasing out. A considerable proportion of the labor force does not work in agriculture, which is a possible reason why the number of household laborers had no significant impact on farmland transfer. The farmland scale positively promoted leasing at the 5% statistical level but had no significant impact on leasing out. This is partly because the farmland scale embodies peasant households’ agricultural production capacity. The stronger the capacity, the stronger the inclination to lease in farmland. With the improvement of agricultural mechanization facilitating part-time business, small-scale farmers do not necessarily lease out their only farmland. The number of plots owned by farmers significantly inhibited the lease in of farmland at the 5% statistical level and also promoted farmland lease-out at the 5% level. This result is similar to the distance between the two plots farthest from the farmland transfer. These two results indicate that farmland fragmentation is important in restricting farmland transfer. The impact of the agricultural machinery on farmland transfer was similar to that of farmland, which promotes farmland lease-in at the 10% statistical level and inhibits farmland lease-out at the 5% level. The improvement of agricultural social services has greatly reduced the difficulty of farming and significantly inhibited lease-out at the 5% statistical level. In the dimension of peasant personal indicators, only medical expenditure significantly promoted the leasing out of farmland at a statistical level of 10%. One possible reason is that medical expenditures negatively correlate with health, and farmers troubled by health problems are more inclined to lease out their farmland.

### 3.2. Robustness Test

To carry out HSFC, a series of processes—such as unified government planning, project bidding, and financial allocation—are needed, which are strongly led by the government. Therefore, implementing HSFC is strongly exogenous, and there is no endogenous problem with the reverse impact of farmland transfer on HSFC [23]. Nevertheless, there is a potential problem of self-selection because there may be some non-randomness factors influencing the implementation of the HSFC, such as the local financial situation and the degree of attention paid by the local government. To effectively deal with the problem of self-selection in sample selection, we learned from existing research [23] and used propensity score matching (PSM) using nearest neighbor matching, kernel matching, and radius matching for estimation. The results show that the treatment effects of HSFC on farmland lease-in are significantly positive, while the treatment effects on lease-out are negative (Table 3). This finding is consistent with the results of the benchmark regression, which means that the above results are robust and reliable.

### 3.3. Moderating Effect of Farmland Fragmentation

Highly finely fragmented farmland is the basic pattern of China’s farmland, which is mainly manifested in two aspects: first, farmers have several plots of farmland, and second, the plots are scattered and far apart. At present, scattered and fragmented distribution is one of the main problems faced by agricultural production and management in different countries and regions of East Asia, including China. During the field survey in Shandong Province, we found that, generally, there are 2–3 farmland plots for households who do not increase population, and 4–6 plots for those who increase. According to some villagers, the smallest of the plots can only grow two rows of corn and is far away from other plots. Therefore, to investigate the impact of HSFC on farmland transfer under different fragmentation conditions, we constructed two groups of interaction terms: the interaction term of HSFC with the number of plots and another interaction of HSFC with the farthest plot distance. The regression results show that HSFC significantly promotes farmland lease at the 5% statistical level. Still, the interaction coefficient of HSFC with the number of plots and the farthest plot distance is significantly negative at the 10% statistical level (Table 4). This indicates that although HSFC can promote farmland lease-in, this role is weakening with increased farmland fragmentation. From the perspective of lease-out, HSFC significantly inhibited farmland lease-out at the 5% level, but both interaction terms were significantly positive at the 10% level (Table 4). This attests that increasing fragmentation weakens the inhibition effect above. Overall, although HSFC is conducive to improving agricultural yield per unit area and raising comparative income, the degree of farmland fragmentation will increase the cost of agricultural production. This dilutes the mitigation effect of HSFC on low agricultural comparative income, which is not conducive to improving farmers’ demand for farmland, and, thus, is not conducive to farmland transfer.

### 3.4. Heterogeneity Analysis

In 2021, the urbanization rate of Shandong Province was approximately 63.94%, an increase of approximately 64% compared with 39% in 2001, meaning, in the past 20 years, a population of 20 million has been transferred from rural areas to cities. Labor transfer is relatively common in Shandong Province, and the existing research shows that when it crosses the threshold, labor transfer is an important factor affecting farmland transfer [23]. Therefore, this study investigated whether the impact of HSFC on farmland transfer varied according to the degree of labor transfer, measured by the proportion of completely non-agricultural labor. A high degree of transfer occurs when the proportion of completely non-agricultural labor is higher than the average. On the contrary, a low degree occurs when it is lower than the average. The results of the heterogeneity analysis show that for a low-degree transfer, HSFC significantly promoted farmland lease-in at the 1% statistical level and significantly inhibited lease-out (Table 5). The impact of HSFC on lease-in and lease-out for high-degree transfers was not significant (Table 5). The possible reason for the above difference is that for peasant households with low degree transfers, farming income is a matter of great account, so they are more sensitive to the promotion of farmland value brought by HSFC. On the contrary, households with high degree transfers are less sensitive to farmland value since farming income is inappreciable, even if the HSFC improves farmland value.

## 4. Discussion

In 2022, Chen and Hong also explored the impact of HSFC on farmland transfer with Jiangxi Province as the sample using different variables from those in this study. Jiangxi Province is located in central China and is home to only 2.1% of the country’s farmland; both its HSFC completion rate and farmland transfer rate are only 11%. Accordingly, that study’s sample size and diversity are extremely limited, which weakens the reliability of its conclusions. To make it convenient to compare with the findings about the influence of HSFC on farmland transfer in Jiangxi Province, the general idea and framework of Chen and Hong [23] were followed. Meanwhile, this paper is significantly different from Chen and Hong’s in some key variables. For example, social relationship is an important potential variable that affects the behavior of farmland transfer. Chen and Hong [23] measured the social relationship of farmers by the number of friends in their WeChat account, while ignoring that the middle-aged and elderly are the main force of agricultural production in China nowadays, and many of them do not use WeChat. In contrast, this study used human relationship expenditure to measure social relations. When calculating the degree of farmland fragmentation, Chen and Hong [23] only considered the number of plots but ignored the scattered distribution of plots as an important manifestation of the fragmentation problem. In contrast, this paper comprehensively considered the numerous and scattered plots involved in the problem of farmland fragmentation. In addition, social service utilization and the health status of farmers objectively measured by medical expenses are all factors that potentially influence farmland transfer; compared with Chen and Hong’s neglect [23], this study set them as control variables. Additionally, this paper also used different methods of robustness testing and exploring the moderating effect as well as heterogeneity analysis, which makes the conclusion more reliable. From the findings, Chen and Hong’s study also confirmed that HSFC can promote farmers to lease in farmland, while inhibiting their enthusiasm to lease out. Compared with Shandong Province, which is dominated by plains, Jiangxi Province is dominated by mountainous and hilly terrain; the two studies with different provinces as samples reached a similar conclusion, suggesting its universality to a certain extent.

## 5. Conclusions

Based on an analysis of 660 questionnaires from five counties located in different regions of Shandong Povince, China using the binary probit model, this study empirically analyzed the impact of government-led HSFC on market-oriented farmland transfers. The results show that the government-led HSFC significantly impacted market-oriented farmland transfer, which significantly promoted farmland lease-in while inhibiting lease-out. Based on the robust research results, the hypocrisies H1 that HSFC can promote farmers to lease in farmland and H2 that HSFC can restrain farmers from leasing out their farmland were confirmed. Furthermore, farmland fragmentation played a significant moderating role in the above impact, illustrated by the fact that improved farmland fragmentation does not promote HSFC in the context of farmland lease-in. Instead, it effectively alleviates the inhibitory effect of HSFC on farmland lease-out. Finally, the impact demonstrates significant heterogeneity in labor transfer. The HSFC can significantly promote farmland lease-in and inhibit lease-out for households with a low degree of labor transfer. In contrast, the above effect is not significant for households with a high degree of labor transfer.

Overall, the conclusions of this study have the following implications for promoting government-led HSFC and market-oriented farmland transfers. First, the HSFC should be strengthened. HSFC will help improve comprehensive agricultural productivity and consolidate food security. Additionally, it significantly raises the value of farmland and encourages large and influential farmers to lease in more farmland. This will surely help to achieve the goal of the moderate-scale operation. Second, the elimination of farmland fragmentation and HSFC should be promoted. The findings in this study show that land fragmentation will greatly reduce the promotion of HSFC on farmland lease-in and alleviate the inhibition of lease-out, which is not conducive to promoting moderate-scale operations. Meanwhile, farmland fragmentation hinders the improvement of agricultural mechanization and is an important factor restraining China’s agricultural productivity. In this context, the favorable opportunity to promote HSFC should be taken advantage of to promote the relocation and reorganization of scattered plots to solve the historical problem of farmland fragmentation. Third, support for large, professional farmers should be increased. This study revealed that the positive impact of HSFC on farmland lease-in is more significant among households with a low degree of labor transfer. In contrast, it is not significant among households with a high degree of labor transfer. Therefore, the government should increase support for large and professional farmers, which is conducive to giving full play to their advantages in agricultural productivity and instrumental in encouraging them to lease in more farmland, to realize moderate-scale operations as soon as possible.

Although in-depth research has been carried out, there are still limitations in this paper. Firstly, Shandong Province is relatively developed along the eastern coast. Although the terrain is diverse, it is mainly plain. The research results obtained by taking Shandong Province as a sample may be different from those of major backward provinces in central and western China. Due to the COVID-19 epidemic, some counties and villages in Shandong Province were on lockdown and were automatically excluded from the sampling. While the completion rate of HSFC, farmland transfer rate, urbanization rate, and local conditions and customs in different counties and villages are similar in close regions, the above exclusion had little impact on sample diversity; the sample bias caused theoretically cannot reflect the overall situation of Shandong Province, and thus affects the accuracy of estimation results. In terms of the sample size, although 660 questionnaires are sufficient to construct cross-sectional data for empirical analysis, considering the huge population of farmers in Shandong Province, there is still room for expansion of sample size. Secondly, variable settings can be further optimized. For example, household income and the proportion of male labor force may affect farmland transfer, but they were ignored in this study. In the follow-up study, a larger cross-regional and multi-provincial field survey can be carried out to obtain more diverse data. Meanwhile, more scientific and comprehensive variables and robustness testing methods can be employed to obtain more dependable conclusions in the future.

## Figures and Tables

**Table 1:** Descriptive Statistical Analysis.

Variables	Description of Variables	Max	Min	MV	SD
**Dependent Variable**					
Whether there was farmland leasing in	Yes = 1; No = 0	1	0	0.09	0.22
Whether there was farmland leasing out	Yes = 1; No = 0	1	0	0.35	0.39
**Main Independent Variable**					
Whether high-standard farmland has been constructed	Yes = 1; No = 0	1	0	0.69	0.58
**Control Variables**					
Village location	Distance from village committee to county town bus station (km), logarithm	3.68	0.69	3	1.27
Village topography	plain = 1; hill = 2; mountain = 3	3	1	1.47	0.79
Amount of household labor	Total adult household labor force	6	1	2.3	0.95
Proportion of completely non-agricultural labor	Proportion of completely non-agricultural labor in total	0.8	0	0.24	0.18
Total scale of farmland	Total scale of Household farmland area (MU), logarithm	5.64	0.08	1.71	1.16
Number of plots	Number of plots of household-operated farmland	6	1	3.39	1.11
Farthest plots distance	The farthest distance between plots of farmland managed by households. Within 0.5 km = 1; 0.5–1 km = 2; Over 1 km = 3	3	1	1.98	0.91
Amount of farm machinery *	Number of farm machinery owned by household	6	0	1.98	1.04
Social service utilization	The frequency of using social services. 1 = never use; 2 = occasionally; 3 = often; 4 = very often	4	1	2.65	2.08
Human relationship expenditure	Less than 100 yuan/year = 1; 100–500 yuan/year = 2; 500–2000 yuan/year = 3; Over 2000 yuan/year = 4	4	1	2.84	1.19
Age	Actual age of individual farmer	67	21	53.37	13.98
Educational level	Primary school and below = 1; Junior high school = 2; High school = 3; Junior college or below = 4	4	1	1.92	0.98
Medical expenses	Less than 100 yuan/year = 1; 100–1000 yuan/year = 2; 1000–5000 yuan/year = 3; Above 5000 yuan/year = 4	4	1	1.86	0.57

* Includes large and small tractors, land leveling machines, plows, cultivators, micro-cultivators, transplanters, seeders, threshers, water pumps, combine harvesters, roller shutters, etc.

**Table 2:** Result of benchmark regression.

Variable	Lease-In (LI)		Lease-Out (LO)	
	Model 1	Model 2	Model 3	Model 4
HSFC	0.984 *** (0.221)	0.713 *** (0.251)	−0.474 ** (0.232)	−0.448 * (0.234)
Village location		0.021 (0.073)		−0.035 (0.084)
Village topography		−0.232 (0.523)		0.301 (0.411)
Amount of household labor		0.122 (0.198)		−0.432 (0.511)
Proportion of completely non-agricultural labor		−0.121 * (0.084)		0.228 ** (0.331)
Total scale of farmland		0.352 ** (0.264)		−0.209 (0.182)
Number of plots		−0.031 ** (0.033)		0.029 ** (0.034)
Farthest plots distance		−0.128 * (0.089)		0.232 ** (0.081)
Amount of farm machinery *		0.527 * (0.315)		−0.444 ** (0.365)
Social service utilization		0.437 (0.218)		−0.525 ** (0.333)
Human relationship expenditure		−0.009 (0.016)		0.011 (0.031)
Age		−0.056 (0.013)		0.007 (0.006)
Educational level		−0.032 (0.019)		0.025 (0.011)
Medical expenses		−0.222 (0.236)		0.321 * (0.131)
County		control		control
Constant	−1.327 *** (0.092)	1.335 ** (0.809)	0.346 *** (0.078)	0.784 *** (0.598)
Pseudo R^2^	0.068	0.215	0.121	0.114

***, **, * respectively indicate significance at the 1%, 5%, and 10% levels.

**Table 3:** Result of robustness test.

Farmland Transfer	Matching Method	HSFC (Yes)	No HSFC (No)	Average Treatment Effects on Treated	Standard Error	T Value
Lease-in (LI)	Nearest Neighbor Matching	0.391	0.196	0.196	0.109	1.84 *
	Kernel Matching	0.373	0.186	0.189	0.090	2.14 **
	Radius Matching	0.391	0.111	0.282	0.086	3.34 ***
Lease-out (LO)	Nearest Neighbor Matching	0.198	0.455	−0.255	0.122	−2.06 **
	Kernel Matching	0.198	0.355	−0.155	0.072	−2.09 *
	Radius Matching	0.198	0.354	−0.154	0.072	−2.08 **

***, **, * respectively indicate significance at the 1%, 5%, and 10% levels.

**Table 4:** Result of moderating effect estimation.

Variable	Lease-In (LI)	Lease-Out (LO)
HSFC	1.115 ** (0.438)	−0.852 ** (0.429)
Number of plots (NP)	−0.085 * (0.072)	0.058 * (0.037)
Farthest plots distance (FPD)	−0.225 * (0.127)	0.372 * (0.272)
HSFC × NP	−0.511 * (0.224)	0.335 * (0.199)
HSFC × FPD	−0.656 * (0.309)	0.452 * (0.266)
Control variable	control	control
Constant	−1.898 *** (0.922)	−453 *** (0.191)
Pseudo R^2^	0.167	0.025

***, **, * respectively indicate significance at the 1%, 5%, and 10% levels.

**Table 5:** Result of heterogeneity analysis.

Variable	Lease-In (LI)		Lease-Out (LO)	
	Low Degree	High Degree	Low Degree	High Degree
HSFC	0.389 *** (0.227)	0.229 (0.186)	−0.132 * (0.315)	−0.625 (0.771)
Control variable	Control	Control	Control	Control
Constant	−1.711 (1.032)	−1.554 * (2.221)	−0.112 ** (0.141)	−0.212 ** (0.091)
Pseudo R^2^	0.167	0.338	0.056	0.291

***, **, * respectively indicate significance at the 1%, 5%, and 10% levels.

## Data Availability

Data available on request due to restrictions e.g., privacy or ethical.

## References

[1] Tan R., Wang R., Heerink N. (2020). Liberalizing rural-to-urban construction land transfers in China: Distribution effects. China Econ. Rev..

[2] Hardie I. (1984). Comparative rents for farmland and timberland in a subregion of the south. J. Agric. Appl. Econ..

[3] Gray J. (2010). The comparative sociology of south africa1. S. Afr. J. Econ..

[4] Noumir A., Langemeier M. (2022). Risk and return of heterogenous farmland locations and qualities. Int. Food Agribus. Manag. Rev..

[5] Schoneveld C. (2014). The geographic and sectoral patterns of large-scale farmland investments in sub-Saharan Africa. Food Policy.

[6] Bélair J. (2021). Farmland investments in Tanzania: The impact of protected domestic markets and patronage relations. World Dev..

[7] Takeshima H., Nin-Pratt A., Diao X. (2013). Mechanization and agricultural technology evolution, agricultural intensification in sub-saharan africa: Typology of agricultural mechanization in Nigeria. Am. J. Agric. Econ..

[8] Madsen J., Islam M. (2016). Exploring the widening food gap: An international perspective. Agric. Econ..

[9] Manoli G., Bonetti S., Scudiero E., Teatini P. (2013). Monitoring and Modeling Farmland Productivity Along the Venice Coastland, Italy. Procedia Environ. Sci..

[10] Motosugi A. (2003). The Structure and Movement of the Farmland and Rural Improvement Project Budget under the Agriculture Basic Law. Phys. Lett. B.

[11] Lin Q., Dai X., Cheng Q., Lin W. (2022). Can Digital Inclusive Finance Promote Food Security? Evidence from China. Sustainability.

[12] Bolton M., Bamford R., Blackburn C., Cromarty J., Eglington S., Ratcliffe N., Sharpe F., Stanbury A., Smart J. (2011). Assessment of simple survey methods to determine breeding population size and productivity of a plover, the Northern Lapwing Vanellus vanellus. Wader Study Group Bull..

[13] Zhao Y., Sun X. (2022). Whether the construction of high standard farmland can help promote the cultivation of new vocational farmers: Evidence from villages. Rural Econ..

[14] Wang Y., Li G., Wang S., Zhang Y., Li D., Zhou H., Yu W., Xu S. (2022). A Comprehensive Evaluation of Benefit of High-Standard Farmland Development in China. Sustainability.

[15] Xia Q., Li L., Zhang B., Dong J. (2022). Nonlinear Influence of Land-Use Transition on Carbon Emission Transfer: A Threshold Regression Analysis of the Middle Reaches of the Yangtze River in China. Land.

[16] Marks-Bielska R. (2021). Conditions underlying agricultural land lease in Poland, in the context of the agency theory. Land Use Policy.

[17] Aikaeli J., Markussen T. (2022). Titling and the value of land in Tanzania. J. Int. Dev..

[18] Deininger K. (2011). Forum on global land grabbing: Challenges posed by the new wave of farmland investment. J. Peasant Stud..

[19] Qiu T., Shi X., He Q., Luo B. (2021). The Paradox of Developing Agricultural Mechanization Services in China: Supporting or Kicking out Smallholder Farmers?. China Econ. Rev..

[20] Tang H., Liu J., Dai X., Zhang Y., He W., Yin Q., Huang F., Ran R., Liu Y. (2022). Household Groups’ Land Use Decisions Investigation Based on Perspective of Livelihood Heterogeneity in Sichuan Province, China. Int. J. Environ. Res. Public Health.

[21] Akram-Lodhi A. (2012). Contextualising land grabbing: Contemporary land deals, the global subsistence crisis and the world food system. Can. J. Dev. Stud..

[22] Sklenicka P., Janovska V., Salek M., Vlasak J., Molnarova K. (2014). The Farmland Rental Paradox: Extreme land ownership fragmentation as a new form of land degradation. Land Use Policy.

[23] Chen J., Hong W. (2022). Does the construction of high standard farmland promote the transfer of agricultural land?. J. Zhongnan Univ. Econ. Law.

[24] Zhang L., Jiang Q. (2019). Policy suggestions for guiding the orderly circulation of rural land management rights. Econ. Rev..

[25] Li X., Li G. (2014). Marketization of rural land transfer: Good rural model and its enlightenment. J. Hunan Agric. Univ. Soc. Sci. Ed..

[26] Yan Y., Cui Q. (2022). Research on the “core edge” relationship of urban agglomeration land price. J. Hebei Univ. Econ. Trade.

[27] Ohajianya D.O., Okwara M.O., Ugwu J.N., Tim Ashama A., Mbah R.O., Dike N.F. (2016). Productivity of Farmland Values in Food Crop Production in the Natural Disasters Prone Areas of Imo State, Nigeria. Int. J. Sustain. Agric. Res..

[28] Obasi P. (2013). Factors Affecting Agricultural Productivity among Arable Crop Farmers in Imo State, Nigeria. Am. J. Exp. Agric..

[29] Bhattarai M., Singh G., Takeshima H., Shekhawat R., Diao X., Takeshima H., Zhang X. (2020). Farm machinery use and the agricultural machinery industries in India: Status, evolution, implications, and lessons learned. An Evolving Paradigm of Agricultural Mechanization Development: How Much Can Africa Learn from Asia?.

[30] Awasthi K. (2014). Socioeconomic determinants of farmland value in India. Land Use Policy.

[31] Huang H., Miller G., Sherrick B., Gómez M. (2006). Factors Influencing Illinois Farmland Values. Am. J. Agric. Econ..

[32] Lehn F., Bahrs E. (2018). Analysis of factors influencing standard farmland values with regard to stronger interventions in the German farmland market. Land Use Policy Int. J. Cover. All Asp. Land Use.

[33] Kropp J., Peckham G. (2015). US agricultural support programs and ethanol policies effects on farmland values and rental rates. Agric. Financ. Rev..

